# Histone Deacetylase Inhibitors Are Protective in Acute but Not in Chronic Models of Ototoxicity

**DOI:** 10.3389/fncel.2017.00315

**Published:** 2017-10-24

**Authors:** Chao-Hui Yang, Zhiqi Liu, Deanna Dong, Jochen Schacht, Dev Arya, Su-Hua Sha

**Affiliations:** ^1^Kresge Hearing Research Institute, Department of Otolaryngology, University of Michigan, Ann Arbor, MI, United States; ^2^Department of Otolaryngology, Kaohsiung Chang Gung Memorial Hospital, Chang Gung University College of Medicine, Kaohsiung, Taiwan; ^3^Department of Pathology and Laboratory Medicine, Medical University of South Carolina, Charleston, SC, United States; ^4^Department of Chemistry, Clemson University, Clemson, SC, United States

**Keywords:** ototoxicity, HDAC inhibitors, prevention of aminoglycoside-induced hearing loss, modification of histone acetylation, acute and chronic animal models

## Abstract

Previous studies have reported that modification of histones alters aminoglycoside-induced hair cell death and hearing loss. In this study, we investigated three FDA-approved histone deacetylase (HDAC) inhibitors (vorinostat/SAHA, belinostat, and panobinostat) as protectants against aminoglycoside-induced ototoxicity in murine cochlear explants and *in vivo* in both guinea pigs and CBA/J mice. Individually, all three HDAC inhibitors reduced gentamicin (GM)-induced hair cell loss in a dose-dependent fashion in explants. *In vivo*, however, treatment with SAHA attenuated neither GM-induced hearing loss and hair cell loss in guinea pigs nor kanamycin (KM)-induced hearing loss and hair cell loss in mice under chronic models of ototoxicity. These findings suggest that treatment with the HDAC inhibitor SAHA attenuates aminoglycoside-induced ototoxicity in an acute model, but not in chronic models, cautioning that one cannot rely solely on *in vitro* experiments to test the efficacy of otoprotectant compounds.

## Introduction

Aminoglycoside antibiotics continue to be indispensable drugs for treatment of acute infections and for specific indications such as treatment of tuberculosis or Pseudomonas infections in patients with cystic fibrosis, owing to their broad antibacterial spectrum and efficacy against resistant bacterial strains. However, their use has been limited due to ototoxic and nephrotoxic side effects. A pathological feature of aminoglycoside-induced ototoxicity is loss of mechanosensory hair cells in the inner ear, resulting in hearing loss and vestibular dysfunction. Since mammalian cochlear sensory hair cells lack the ability to regenerate, the loss of or damage to sensory hair cells is permanent. Although there have been efforts to find potential therapies to mitigate these side effects, no established clinical therapies for prevention or amelioration are yet available.

Gene expression can be modulated by epigenetic changes in response to physiological and metabolic demand or to environmental stimuli. For example, gene expression is silenced by condensing chromatin, methylating DNA, or deacetylating histones. Conversely, histones may be acetylated and chromatin will unravel to facilitate gene transcription. Two types of enzymes can modulate the acetylation status of histones: (1) Histone acetyltransferases (HATs) add acetyl groups to lysine residues on the histone tails, facilitating the binding of transcription factors to nucleosome DNA and activate transcription. (2) Histone deacetylases (HDACs) remove the acetyl groups, promoting chromatin condensation, and reduce transcription (Marks et al., 2001).

An imbalance of histone acetylation has been linked to a variety of diseases. For example, deficient histone acetylation is found in cancer and progressive neurodegenerative diseases, such as Parkinson and Alzheimer’s diseases (Falkenberg and Johnstone, 2014). Our previous research has found that aminoglycoside antibiotics increased HDAC1, HDAC3 (class I HDACs) and HDAC4 (class II HDAC) in outer hair cells (OHCs) and reduced histone acetylation. The HDAC inhibitors trichostatin A and sodium butyrate rescued OHCs during acute aminoglycoside toxicity (Chen et al., 2009). These two drugs are highly toxic and rarely used in the clinic. However, in recent years, several less toxic HDAC inhibitors have been tested in clinical trials or are already approved by the FDA for the treatment of cancer. Among these, SAHA (vorinostat) attenuated hearing loss in mice caused by treatment with the combination of kanamycin (KM) and furosemide or by noise (Layman et al., 2015; Wen et al., 2015; Chen et al., 2016).

In order to provide basic information on the potential of HDAC inhibitors as therapeutic protectants, we evaluated three of these inhibitors (SAHA, belinostat, and panobinostat), which specifically inhibit HDAC subtypes I, II, and IV, for their likely protective effects during aminoglycoside challenge to organ of Corti explants. We then assessed the protective effects of SAHA *in vivo* both in guinea pigs and CBA/J mice in chronic hearing-loss models that we have previously established (Sha et al., 2001; Wu et al., 2001).

## Materials and Methods

### Animals

For breeding, sexually mature male (6-week-old) and female (8-week-old) CBA/J mice were purchased from Harlan Sprague Dawley Incorporation (Indianapolis, IN, USA). For KM *in vivo* trials, male CBA/J mice at 4 weeks of age were purchased from Jackson Laboratory (Bar Harbor, ME, USA). Male Hartley guinea pigs (200–250 g) were purchased from Charles River (Wilmington, MA, USA). All animals were kept at 22 ± 1°C under a standard 12-h light/12-h dark schedule and had free access to water and a regular mouse (Harlan 2918) or guinea pig diet (#5025; 18.5% protein; Purina, St. Louis, MO, USA). All experimental protocols and all compounds used were approved either by the University of Michigan Committee on the Use and Care of Animals (UCUCA) or the Institutional Animal Care & Use Committee at the Medical University of South Carolina (MUSC). Animal care was under the supervision of the University of Michigan’s Unit for Laboratory Animal Medicine (ULAM) or under the supervision of the Division of Laboratory Animal Resources at MUSC.

### Organotypic Cultures of Post-Natal Murine Organ of Corti

The culture procedures have been described in detail (Chen et al., 2009). In brief, postnatal day 3 (p3) CBA/J pups were euthanized after antisepsis using 70% ethanol. Inner ears were extracted from surrounding tissue and immersed in cold Hank’s balanced salt solution. The lateral wall tissues (stria vascularis and spiral ligament) and the auditory nerve bundle were micro-dissected from the organ of Corti. The explants were placed onto a prepared culture dish containing a 15-μL polymerized drop of rat tail collagen in 1 mL of culture medium consisting of Basal Medium Eagle, 1% serum-free supplement (Invitrogen, Carlsbad, CA, USA), 1% bovine serum albumin, 5 mg/mL glucose, and 10 U/mL penicillin G. After 4 h of incubation (37°C, 5% CO_2_), an additional 1 mL of medium was added to submerge the explants.

### Treatment of the Explants

Explants were incubated for 2 days to recover from dissection stress before administering HDAC inhibitors and gentamicin (GM; Sigma-Aldrich Co., St. Louis, MO, USA). The medium was then exchanged for new medium containing a final concentration of 4.5 μM GM with or without various concentrations of the HDAC inhibitors and incubated for 72 h. The stock solutions with 95 mM of SAHA/vorinostat (Cayman Chemical, Ann Arbor, MI, USA), 100 mM of belinostat (Selleckchem, Houson, TX, USA), and 100 mM of panobinostat (Cayman Chemical), were dissolved in 100% dimethyl sulfoxide (DMSO) and stored at −20°C. GM solution was made fresh from powder in culture medium at 0.2 mM and then diluted to the final concentration for each experiment.

### Evaluation of Ototoxicity by Hair Cell Counts in Explants

Explants were fixed with 4% paraformaldehyde overnight at 4°C and permeabilized for 30 min with 3% Triton X-100 in phosphate-buffered saline (PBS) at room temperature (22–24°C). The specimens were then washed three times with PBS and incubated with rhodamine-phalloidin (Invitrogen) at a 1:100 dilution for 60 min at room temperature (22–24°C). After rinsing in PBS, the specimens were mounted on a slide with Gel-Mount™ (BioMeda Corp., Foster City, CA, USA). The phalloidin-stained stereociliary bundles and circumferential F-actin rings on the cuticular plate of OHCs allowed the determination of cells that were present or missing. A clear “V” shape of stereocilia indicates the presence of OHCs. If the “V” shape is not visualized, we accept this as a damaged hair cells. Cell populations were assessed on a Leitz Orthoplan upright microscope equipped for epifluorescence, using a 50× oil-immersion objective. The right objective had a 0.19-mm calibrated scale imposed on the field for reference and all three rows of OHCs were oriented longitudinally within each 0.19-mm frame. Each successive 0.19-mm field was evaluated for the absence of OHCs beginning from the apex and moving down the organ of Corti to the base. The percentage of OHC loss was calculated.

### Drug Administration to CBA/J Mice *in Vivo*

Male CBA/J mice at the age of 5 weeks received subcutaneous (SQ) injections of KM at 700 mg base/kg (dissolved in saline) twice per day (AM and PM). SAHA for administration was dissolved in 100% DMSO for stock solutions of 50 mg/mL and diluted with saline immediately before intraperitoneal (IP) injection, for a final dose of 50 mg/kg, given twice per day for 15 days. The concentration of DMSO vehicle control was 1 mL/kg body weight. All solutions were filtered with a syringe filter before injection. Body weight was measured before each injection.

### Experimental Groups and Drug Administration to Pigmented Guinea Pigs *in Vivo*

Pigmented male guinea pigs initially weighing 200–250 g from Charles River were used in the *in-vivo* study. Experiments were begun 1 week after the guinea pigs arrived. To select safe doses of SAHA for protective studies, we conducted two separate sets of experiments to assess serum platelet concentrations. In the first set, pigmented guinea pigs received daily IP injections of SAHA at lower doses (LD; 2 mg/kg and 5 mg/kg) concurrent with SQ injections of 120 mg GM base/kg body weight daily for 2 weeks. In the second study, the higher doses (HD) of SAHA (15 mg/kg and 25 mg/kg) were used. The DMSO concentration was 1.1 g/kg, equal to the DMSO concentration used for the 25 mg/kg concentration of SAHA. Since the highest dose (25 mg/kg/day for 14 days) of SAHA significantly reduced serum platelet concentrations, the doses of SAHA used for the protective studies were 25 mg/kg or less. We then tested the protective ability of SAHA at LD (2 mg/kg and 5 mg/kg) and at HD of SAHA (15 mg/kg and 25 mg/kg) against GM-induced hearing loss concurrent with daily SQ injections of GM (dissolved in saline) at 120 mg base/kg for 14 days. SAHA was dissolved in 100% DMSO for stock solutions of 25 mg/mL and diluted with saline immediately before IP injections. All compound solutions were filtered with syringe filters before injection. Body weights were measured before each injection. If a guinea pig lost ~5% of its body weight, an additional 3–4 mL of saline was administrated subcutaneously.

### Surface Preparations of Guinea Pig Cochlear Epithelia for Hair Cell Counts

The procedures for surface preparations of guinea pig cochlear epithelia were followed as previously described (Sha and Schacht, 1999b). After the last auditory brain stem response (ABR) measurement, guinea pigs from the second group of experiments were deeply anesthetized in a CO_2_ chamber and decapitated. Cochleae were immediately removed and perfused with 4% paraformaldehyde in PBS at pH 7.4 and fixed overnight at 4°C. Micro-dissected surface preparations of the organ of Corti were permeabilized for 30 min with 0.5% Triton X-100 in PBS at room temperature (22–24°C) and stained with rhodamine phalloidin (Invitrogen) at a 1:100 dilution for 60 min at room temperature (22–24°C). After rinsing in PBS, the specimens were mounted on a slide with Gel-Mount™ (BioMeda Corp.). OHC counts were conducted with microscopy as described above for mice.

### Assessment of Guinea Pig Blood Urea Nitrogen (BUN), Creatinine, and Platelet Levels

Blood was obtained by nail clipping after light anesthesia of the guinea pigs by metofane inhalation after the last drug injections. Blood cells were separated by centrifugation at 1000× *g* for 15 min, and sera were stored at −20°C. Blood urea nitrogen (BUN), creatinine and platelet concentrations were determined using a Kodak Ektachem700XR Clinical Chemistry Analyzer (Clinical Products Division, Eastman Kodak, Rochester, NY, USA) and analyzed by Dr. Donald Giacherio, Chemical Pathology Laboratory, University of Michigan.

### Auditory Brainstem Response (ABR)

ABRs were measured 3 days before and 3 weeks after drug treatments. Animals were anesthetized with IP injections of ketamine (100 mg/kg) and xylazine (10 mg/kg) for mice or ketamine (58.8 mg/kg), xylazine (2.4 mg/kg), and acepromazine (1.2 mg/kg) for guinea pigs. Body temperature was maintained near 37°C with a heating pad. ABRs were measured at 12 and 32 kHz for guinea pigs or 8, 16, and 32 kHz for mice in a sound-isolated and electrically shielded booth (Acoustic Systems, Austin, TX, USA). Sub-dermal electrodes were inserted at the vertex of the skull, under the left ear and under the right ear (ground). Tucker Davis Technology System III hardware and SigGen/Biosig software were used to present the stimuli monaurally (15 ms duration tone bursts with 1 ms rise-fall time) through a Beyer earphone attached to a customized plastic speculum inserted into the ear canal. Up to 1024 responses were averaged for each stimulus level. Thresholds (the lowest stimulus level with a reproducible response) were determined by reducing the intensity in 10-dB steps and then in 5-dB steps approaching the threshold until no organized response was detected. Thresholds were evaluated based on ABR wave I. Scores were given by an expert blinded to experimental conditions between the highest stimulus level without a response and the lowest stimulus level where an organized response was observed. All ABR measurements were conducted by the same experimenter.

### Statistical Analysis

Data were analyzed using SYSTAT and GraphPad software for Windows. The group size (*n*) *in vivo* was determined by the variability of measurements and the magnitude of the differences between groups. Statistical methods used include one-way analysis of variance (ANOVA) with Tukey’s multiple comparisons and unpaired *t*-tests. All tests were two-tailed and a *p*-value < 0.05 was considered statistically significant.

## Results

### Toxicity of HDAC Inhibitors in Murine Cochlear Explants

**Figure 1:**
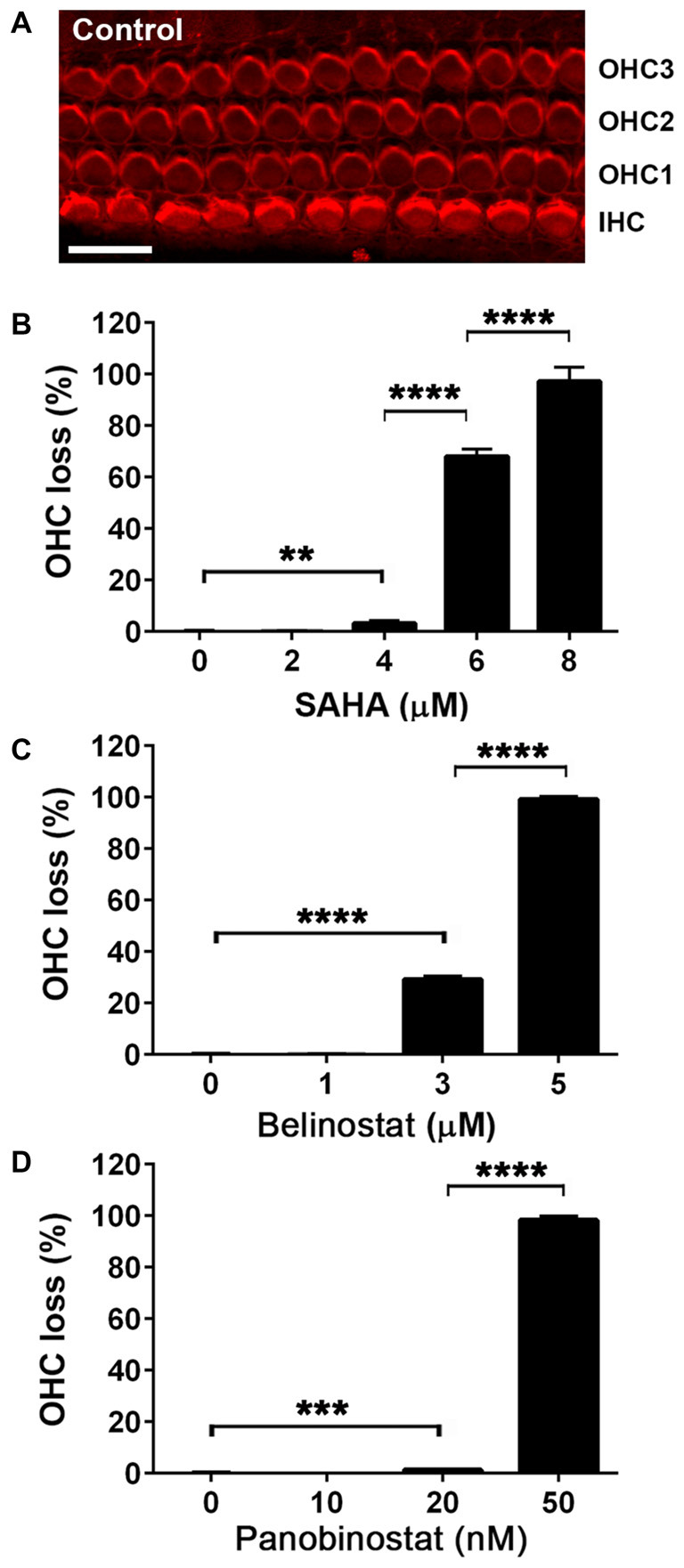
Safety and toxicity of Histone deacetylase (HDAC) inhibitors in organ of Corti explants. **(A)** Image of a control p3 murine explant after culture for 5 days, stained with rhodamine phalloidin (red) to illustrate the preservation of three rows of outer hair cell (OHC) and one row of inner hair cells (IHCs) under the test conditions. Confocal images were taken from the basal turn. Scale bar = 10 μm. **(B–D)** Incubations in the presence of HDAC inhibitors for 72 h damaged hair cells of the basal turn in a dose-dependent manner. Data are presented as means ± SD, *n* = 3 or 4 at each concentration. **p < 0.01, ***p < 0.001, ****p < 0.0001.

In order to evaluate the protective potential of the three HDAC inhibitors on aminoglycoside-induced ototoxicity, we first determined their safety on auditory sensory hair cells using p3 murine organ of Corti explants. Explants were incubated for 2 days for recovery from the stress of dissection, followed by 72 h of incubation with or without inhibitors. Toxic effects were assessed by OHC loss. Control samples showed no loss of cells (Figure 1A) but all three HDAC inhibitors damaged OHCs in a dose-dependent manner. OHCs in the basal turn show changes in the orientation of the stereociliary bundles beginning with concentrations of 4 μM for SAHA, 3 μM for belinostat, and 20 nM for panobinostat. The toxic effects increased significantly with increasing concentrations and loss of OHCs reached 100% at 8 μM of SAHA, 5 μM of belinostat, and 50 nM of panobinostat (*p* < 0.0001). Statistical analysis using one-way ANOVA showed a significant dose-dependence of the toxic effect for SAHA (*F*_(4,12)_ = 840.8, *p* < 0.0001), as well as for belinostat (*F*_(3,9)_ = 11,856, *p* < 0.0001) and panobinostat (*F*_(3,9)_ = 13,972, *p* < 0.0001; Figures 1B–D; for detailed Tukey’s multiple comparison test values see Table 1). The median toxic doses (TD50), defined as the concentrations leading to 50% OHC loss, for SAHA, belinostat and panobinostat were 5.5 μM, 3.5 μM, and 35 nM, respectively.

**Table 1:** Tukey’s multiple comparison test values for data in Figure 1.

HDAC inhibitors	Groups	*p* Value	*P* value summary
SAHA	0 vs. 4 μM	0.0089	**
	0 vs. 6 μM	<0.0001	***
	4 vs. 8 μM	<0.0001	****
	6 vs. 6 μM	0.0004	***
Belinostat	0 vs. 3 μM	<0.0001	****
	3 vs. 5 μM	<0.0001	****
Panobinostat	0 vs. 20 nM	0.0003	***
	20 vs. 50 nM	<0.0001	****

### HDAC Inhibitors Protect Against Gentamicin-Induced Outer Hair Cell Loss in Murine Explants in a Dose-Dependent Manner

**Figure 2:**
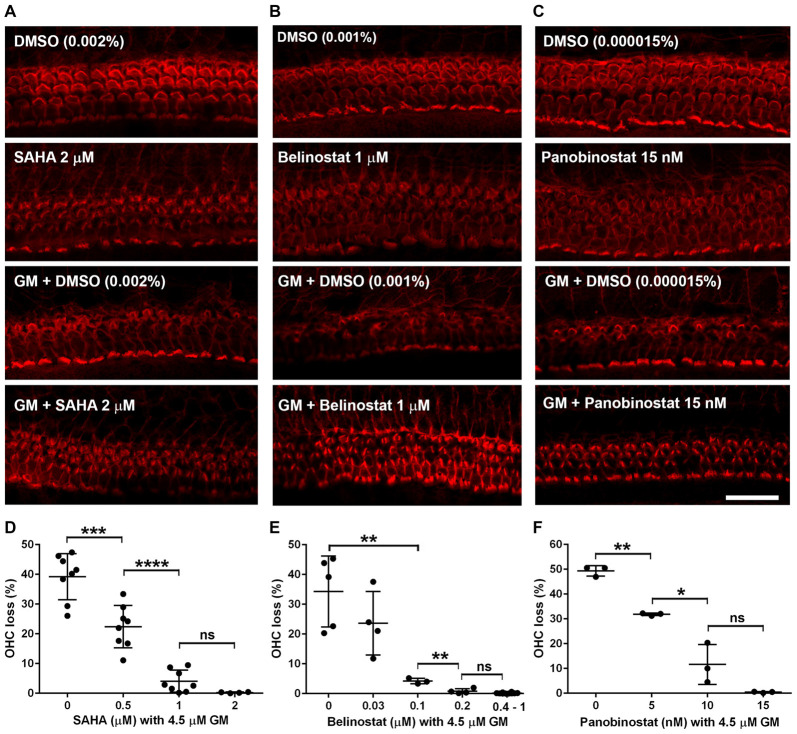
HDAC inhibitors protect against gentamicin (GM)-induced hair cell loss in p3 murine explants. **(A–C)** Representative images show that administration of HDAC inhibitors (**A**: SAHA, **B**: belinostat, **C**: panobinostat) attenuated GM-induced sensory hair cell loss in the basal turn. Scale bar = 20 μm. **(D–F)** Quantification of HDAC inhibitors (**D**: SAHA, **E**: belinostat, **F**: panobinostat) prevented OHC loss from GM in a dose-dependent manner. Data are presented as individual points and means ± SD. *p < 0.05, **p < 0.01, ***p < 0.001, ****p < 0.0001, ns: not significant.

After having established the safe concentration range for the HDAC inhibitors, we explored their efficacy as protective agents against GM-induced OHC loss. In the 72-h incubations of explants, GM with the vehicle alone (DMSO) caused loss of OHCs in a base-to-apex gradient with a 40% overall loss at 4.5 μM. Co-incubation with SAHA (Figure 2A) proved protective, beginning at 0.5 μM SAHA and increasing in efficacy with increased doses, such that 2 μM SAHA completely protected from OHC loss (*F*_(3,24)_ = 59.95, *p* < 0.001; Figure 2D). Similarly, co-administration of belinostat (Figure 2B) or panobinostat (Figure 2C) with GM also reduced GM-induced OHC loss in a dose-dependent manner. GM-induced OHC loss was attenuated by co-administration of 0.1 μM belinostat and was totally prevented by 0.4 μM (*F*_(4,21)_ = 29.2, *p* < 0.001; Figure 2E). Additionally, with administration of panobinostat protection started at 5 nM and OHC loss was completely blocked by 15 nM panobinostat (*F*_(3,8)_ = 81.19, *p* < 0.001; Figure 2F). From the assessment of safe doses and the median effective (protective) doses (ED50) of the three HDAC inhibitors, we calculated the therapeutic index (TI = TD50/ED50) to be highest (safest) for belinostat and lowest for panobinostat (for detailed values see Table 2).

**Table 2:** Therapeutic index (TI) of three HDAC inhibitors in murine explants.

HDAC inhibitors	TD_50_	ED_50_	TI (TD_50_/ED_50_)
Vorinostat	5.5 μM	0.6 μM	9.2
Belinostat	3.5 μM	0.05 μM	70
Panobinostat	35 nM	9 nM	3.9

### SAHA Does Not Prevent Kanamycin-Induced Hearing Loss in Mice *in Vivo*

**Figure 3:**
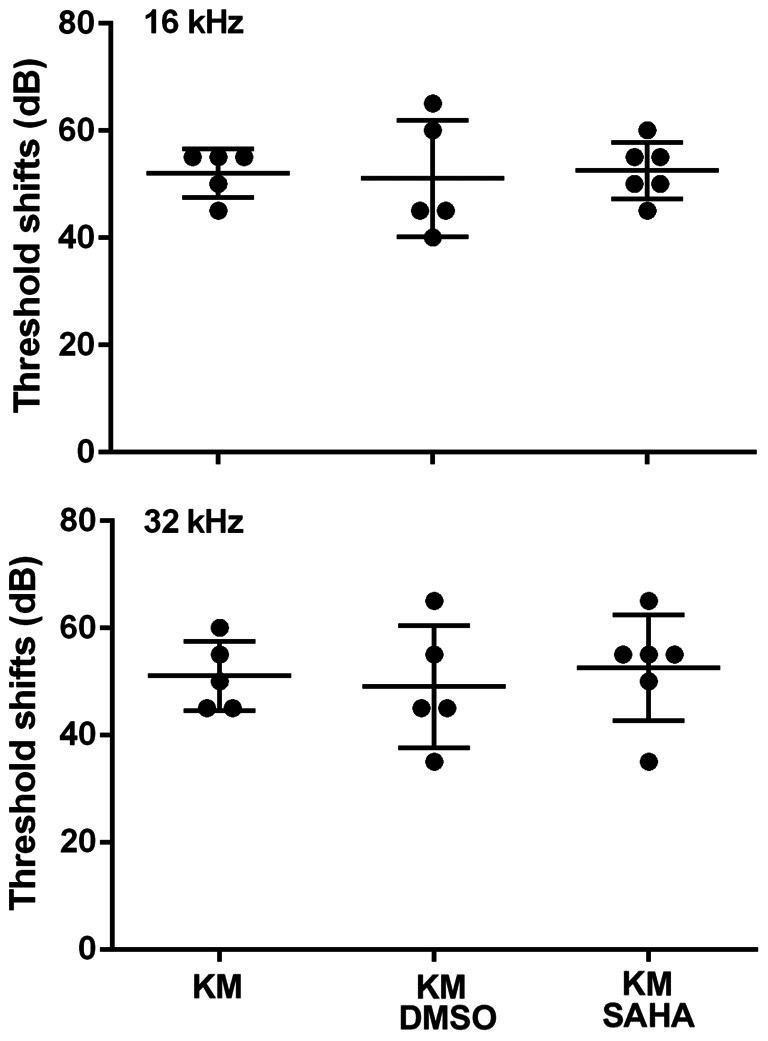
Treatment with SAHA (50 mg/kg twice daily for 15 days) does not attenuate kanamycin (KM, 700 mg base/kg twice daily for 15 days)-induced auditory threshold shifts in CBA/J mice. Data are presented as individual points and means ± SD.

In order to assess the protective potential of SAHA *in vivo* in a chronic ototoxicity model, we tested if SAHA treatment had protective effects against KM (700 mg base/kg body weight twice daily)-induced hearing loss in CBA/J mice. Consistent with our previous studies (Wu et al., 2001), treatment with KM for 15 days caused auditory threshold shifts of an average of 55 dB at 16 kHz (*p* < 0.0001) and 32 kHz (*p* < 0.0001). Treatment with SAHA at 50 mg/kg body weight twice daily did not attenuate KM-induced auditory threshold shifts (Figure 3). Typical ABR waveforms for each treated group are illustrated in Supplementary Figure S1.

### The Effects of SAHA Treatment on Platelet Concentration in Guinea Pigs *in Vivo*

**Figure 4:**
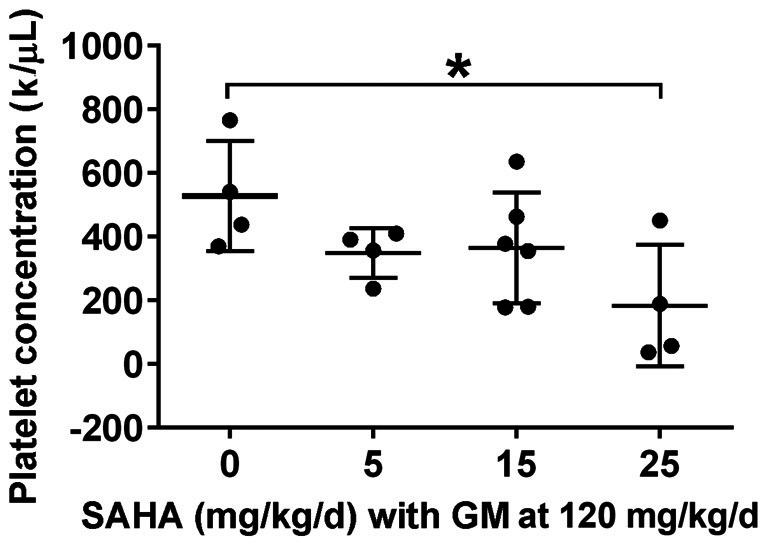
Effects of SAHA treatment on platelet concentration. Treatment with SAHA for 2 weeks led to a decrease in platelet concentration, significant at 25 mg SAHA/kg with 120 mg GM base/kg in guinea pigs. Data are presented as individual points and means ± SD. *p < 0.05.

While the CBA/J mouse is a well-established model for chronic KM-induced ototoxicity, we wished to ascertain whether the lack of protection by SAHA might be species-dependent. We therefore tested if SAHA treatment had protective effects against GM-induced hearing loss in guinea pigs. To find safe doses to be used in guinea pigs *in vivo*, we first assessed serum platelet concentrations. Guinea pigs tolerated injections of SAHA at all concentrations of 5–25 mg/kg body weight concurrent with SQ injections of 120 mg GM base/kg body weight daily for 2 weeks with normal appearing fur and steadily increasing body weight. Blood samples were collected after the last injection. Renal function, assessed by BUN and creatinine (Cr) concentrations, was similar to that of control guinea pigs (detailed values see Table 3). However, the platelet concentration was significantly lower with the highest concentration of SAHA (25 mg/kg; *t*_6_ = 2.682, *p* = 0.037) compared to GM alone treated groups (Figure 4).

**Table 3:** Guinea pigs renal function tests.

Groups	BUN	Cr	*n*
DMSO	17.8 ± 1.6	0.5 ± 0.19	5
SAHA (LD + HD)	17.5 ± 0.7	0.5 ± 0.007	2
GM + DMSO	16 ± 4.5	0.5 ± 0.22	4
GM + SAHA (LD)	17 ± 1.7	0.4 ± 0.06	6
GM + SAHA (HD)	17 ± 3.2	0.4 ± 0.08	5

*LD: 2–5 mg/kg; HD: 15–25 mg/kg*.

### SAHA Does Not Prevent Gentamicin-Induced Ototoxicity in Guinea Pigs *in Vivo*

**Figure 5:**
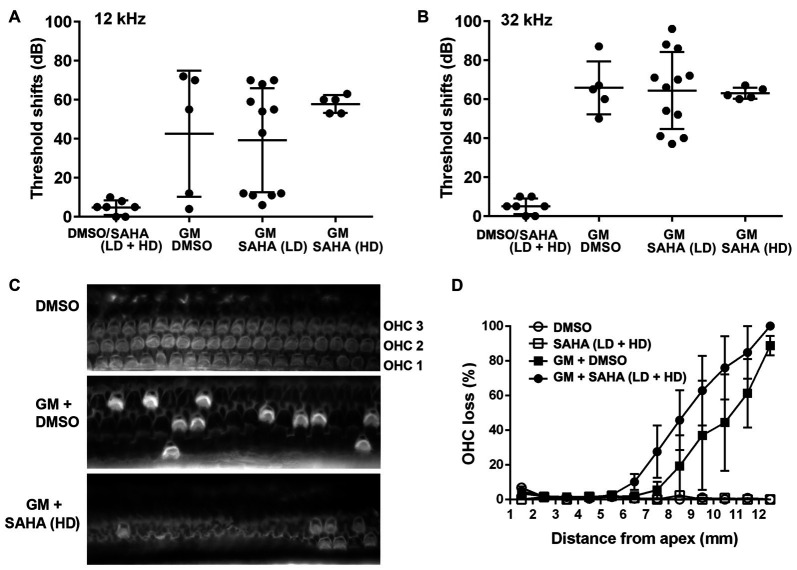
Treatment with SAHA does not protect against GM-induced auditory threshold shifts and hair cell loss in pigmented guinea pigs *in vivo*. **(A,B)** Co-treatment with SAHA at low doses (2–5 mg/kg, LD) or high doses (15–25 mg/kg, HD) did not attenuate GM-induced (120 mg/kg, qd × 15 days) auditory threshold shifts at 12 kHz and 32 kHz. Data are presented as individual points and means ± SD. **(C)** Representative images illustrated OHC loss in the basal turn of surface preparations examined 1 week after 15 days of GM treatment. OHC1, 2 and 3 indicate the three rows of OHCs. Scale bar = 10 μm. **(D)** GM-induced OHC loss was not attenuated by treatment with SAHA in pigmented guinea pigs. Data are presented as means ± SD, *n* = 3–5 at each group.

Based on the experiments to assess safe doses of SAHA, we tested several doses of SAHA below 25 mg/kg body weight against 120 mg GM base/kg body weight daily for 2 weeks in guinea pigs *in vivo*. In agreement with our previous results, GM treatment induced significant auditory threshold shifts at high frequencies (Figures 5A,B) with an average shift of 40 dB at 12 kHz (*t*_10_ = 3.128, *p* = 0.011) and 60 dB at 32 kHz (*p* < 0.0001) and caused significant OHC loss in the basal turn (*F*_(1,4)_ = 8.013, *p* = 0.047; Figures 5C,D). Confirming the results from CBA/J mice, treatment with SAHA at a range of concentrations (LD: 2–5 mg/kg body weight or HD: 15–25 mg/kg body weight) did not attenuate GM-induced auditory threshold shifts at 12 (*F*_(2,19)_ = 0.9708, *p* = 0.397) and 32 kHz (*F*_(2,19)_ = 0.03664, *p* = 0.964) or loss of OHCs (*F*_(1,6)_ = 0.849, *p* = 0.392). DMSO (1.1 g/kg body weight) used as the solvent vehicle for SAHA did not affect GM-induced auditory threshold shifts (Figure 5A).

## Discussion

An intriguing line of evidence suggests that HDAC inhibitors could prove to be an effective modality to attenuate aminoglycoside-induced ototoxicity in acute models. The HDAC inhibitors trichostatin A and butyrate rescued hair cells from GM-induced damage in organotypic cultures of the cochlea (Chen et al., 2009), and butyrate also protected guinea pigs *in vivo* when locally applied to the middle ear (Wang et al., 2015). Systemic SAHA attenuated the acute effects of the ototoxic combination of KM and furosemide (Layman et al., 2015) and the trauma of exposure to excessive noise (Chen et al., 2016). The results presented here extend the palette of potential therapeutics to three clinically available HDAC inhibitors when studied in cochlear explants. *In vivo*, however, SAHA failed to reduce hair cell loss and threshold shifts induced by *chronic* aminoglycoside exposure both in mice and guinea pigs. This *in-vivo* result offers an important caution because the chronic animal model better reflects clinical situations in which aminoglycosides tend to be administered for weeks, or even months in cases like tuberculosis.

In a chronic model the protective capability of the drug must be weighed against its potential side effects. Arguing for their safety, the chosen drugs are either FDA approved for treatment of cutaneous T-cell lymphoma (SAHA) or peripheral T-cell lymphoma (belinostat) or in a phase 3 trial for multiple myeloma (panobinostat). Indeed, although the median effective dose (ED50) varies, GM-induced OHC loss in explants is completely blocked by any of three HDAC inhibitors within non-toxic concentrations. However, as the explant studies also show, all three inhibitors tend to kill hair cells at higher concentrations. Based on the therapeutic index (TI = TD50/ED50), belinostat is the safest among the three HDAC inhibitors and might be selected for further *in-vivo* studies. We chose SAHA, however, based on published *in-vivo* data suggesting SAHA is suitable for prevention of inner ear trauma in mice (Layman et al., 2015; Chen et al., 2016). For example, administration of SAHA for 2 weeks at 100 mg/kg/day showed no ototoxicity in mice and IP injection of SAHA penetrates the mouse inner ear and crosses the blood-labyrinth barrier (Layman et al., 2015). SAHA also crosses the blood-brain barrier in a mouse model of Huntington’s disease (Hockly et al., 2003) and thus can be expected to cross the blood-labyrinth barrier to the inner ear as well. We, therefore, tested SAHA in an established and reliable mouse model of chronic ototoxicity from 700 mg/kg KM twice per day for 2 weeks (Wu et al., 2001). Administration of SAHA at 100 mg/kg/day (dosed as SAHA at 50 mg/kg/twice daily concurrent with KM injections), however, did not prevent KM ototoxicity.

In order to challenge the robustness of our results and to rule out species-specific effects, we tested protection in the guinea pig, another well-established chronic model of GM-induced ototoxicity (Sha and Schacht, 1999b, 2000). In agreement with our previous data, treatment with GM at 120 mg/kg/day for 2 weeks resulted in significant elevations of auditory thresholds. Here again, administration of SAHA at low doses (2–5 mg/kg) or high doses (15–25 mg/kg/day) did not attenuate GM-induced auditory threshold shifts. Since the highest dose (25 mg/kg/day for 14 days) significantly reduced serum platelet concentrations, further increases in the dose of SAHA were considered to have no bearing for translational research.

The difference in the ability of SAHA to protect against inner ear insults in acute and chronic animal models of aminoglycoside-induced ototoxicity could be attributable to the vast differences between models used in the studies. In an acute model, as reported by Layman et al. (2015), the ototoxicity is induced by one SQ dose of KM with one dose of furosemide via IP injection. In contrast, the chronic model of ototoxicity is induced by KM alone via SQ injection for 15 days twice per day. Although SAHA may cross the blood-labyrinth barrier to the inner ear, administration of furosemide or noise exposure may facilitate compounds crossing the blood-labyrinth barrier. Additionally, differences may be owed to the cell death pathways induced, as we previously reported that caspase-independent hair cell death predominates such chronic *in-vivo* models (Jiang et al., 2006). Such disparities may account for why some *in-vivo* models show a protective effect against aminoglycosides while others may not. Furthermore, such difference between acute and chronic models cautions that one cannot rely solely on *in-vitro* experiments to test the efficacy of otoprotectant compounds.

HDACs regulate acetylation of histone proteins at specific arginine and lysine residues, changing chromatin structure and altering gene expression. Acetylation tends to promote gene expression while inner ear insults such as aminoglycoside treatment or noise exposure promote histone deacetylation via HDACs. Among the four classes of mammalian HDACs identified (Dietz and Casaccia, 2010), class II has been recognized as having a pathogenic role in neurodegenerative diseases such as Alzheimer’s disease (Falkenberg and Johnstone, 2014) and in HIV-infected neuronal cells (Atluri et al., 2014). In line with our previous and studies by others, the three HDAC inhibitors used in this study cover an even broader spectrum, acting as inhibitors of HDACs I, II, and VI (Chen et al., 2009, 2016; Layman et al., 2015).

The mechanism of the epigenetic effects of aminoglycosides remains unknown. Since aminoglycoside-induced ototoxicity is well documented to involve oxidative stress (Sha and Schacht, 1999a; Chen et al., 2013), it is reasonable to speculate that a pathway leading to the upregulation of HDAC involves oxidative stress, a condition that can lead to changes in genomic and epigenetic regulation of gene expression (Mikhed et al., 2015). In particular, gene expression mapping after ischemia-induced oxidative stress demonstrated cell-specific upregulation of HDAC 1, 2 and 3 in the brain (Baltan et al., 2011). Additionally, inner ear insults such as traumatic noise exposure induce oxidative stress (Ohlemiller et al., 1999; Yamashita et al., 2004; Yuan et al., 2015) and result in upregulation of HDAC 1, 2 and 3 in the inner ear of CBA/J mice (Chen et al., 2016).

In summary, this study highlights potential disparities between acute and chronic inner ear damage by demonstrating that treatment with the HDAC inhibitor SAHA attenuates aminoglycoside-induced ototoxicity *in vitro* but not *in vivo* in chronic animal models. Furthermore, our results suggest that the protective effects of other HDAC inhibitors against aminoglycoside-induced ototoxicity deserve careful exploration in animal models and more detailed studies, such as study of drug kinetics and permeability through the blood-labyrinth barrier, before these compounds are considered for clinical application.

## Author Contributions

C-HY performed *in vivo* and *in vitro* experiments. ZL conducted *in-vivo* experiments. DD conducted *in-vitro* experiments. JS designed research and commented on the manuscript. DA commented on the manuscript. S-HS designed research, analyzed data and wrote the article.

## Conflict of Interest Statement

The authors declare that the research was conducted in the absence of any commercial or financial relationships that could be construed as a potential conflict of interest.

## Supplementary Material

FIGURE S1 Typical ABR waveforms of CBA/J mice at 16 and 32 kHz. Baseline testing revealed normal ABRs measured before kanamycin (KM) treatment. The baseline thresholds at both 16 and 32 kHz were 25 dB SPL. I, II, III, IV and V indicate ABR waves I, II, III, IV and V. The images for KM, KM plus DMSO, and KM plus SAHA illustrate representative ABR waveforms measured 1 week after the end of 15 days of treatment.

## Abbreviations

BUN: Blood urea nitrogen
Cr: Creatinine
DMSO: Dimethyl sulfoxide
ED50: Median effective dose
H4K8ac: Acetylation of histone H4 at lysine 8
HATs: Histone acetyltransferases
HD: Higher doses
HDACs: Histone deacetylases
IP: Intraperitoneal
LD: Lower doses
SQ: Subcutaneous
TD50: Median toxic dose
TI: Therapeutic index.
