# Correction: Histone deacetylase inhibitors are protective in acute but not in chronic models of ototoxicity

**DOI:** 10.3389/fncel.2026.1819319

**Published:** 2026-04-01

**Authors:** Chao-Hui Yang, Zhiqi Liu, Deanna Dong, Jochen Schacht, Dev Arya, Su-Hua Sha

**Affiliations:** 1 Kresge Hearing Research Institute, Department of Otolaryngology, University of Michigan, Ann Arbor, MI, United States; 2 Department of Otolaryngology, Kaohsiung Chang Gung Memorial Hospital, Chang Gung University College of Medicine, Kaohsiung, Taiwan; 3 Department of Pathology and Laboratory Medicine, Medical University of South Carolina, Charleston, SC, United States; 4 Department of Chemistry, Clemson University, Clemson, SC, United States

**Keywords:** ototoxicity, HDAC inhibitors, prevention of aminoglycoside-induced hearing loss, modification of histone acetylation, acute and chronic animal models

In the originally published article, an incorrect version of **Supplementary Figure S1** (Image 1.TIF) was inadvertently uploaded. Due to an editorial assembly error, the ABR waveforms for the KM (16 kHz) group were mistakenly duplicated and appeared as the waveforms for the KM + DMSO (32 kHz) group. The file has now been replaced.

The original version of this article has been updated.

