# Supplementary figures and images for: Histone Deacetylase Inhibitors Are Protective in Acute but Not in Chronic Models of Ototoxicity
Source: Front Cell Neurosci. 2017 Oct 24;11:315. doi: 10.3389/fncel.2017.00315 (PMC5660723; doi:10.3389/fncel.2017.00315)

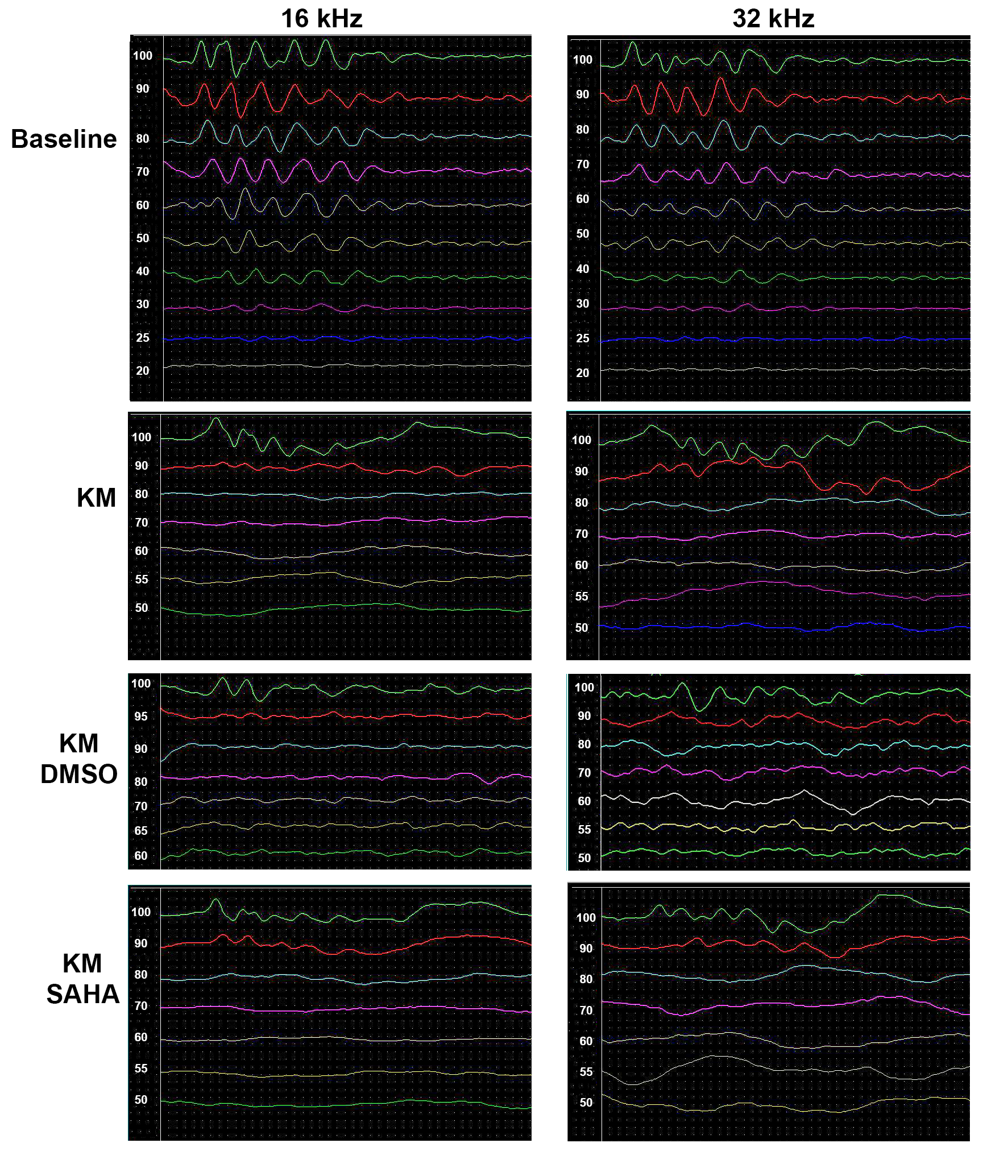

Supplement: FIGURE S1 — Typical ABR waveforms of CBA/J mice at 16 and 32 kHz. Baseline testing revealed normal ABRs measured before kanamycin (KM) treatment. The baseline thresholds at both 16 and 32 kHz were 25 dB SPL. I, II, III, IV and V indicate ABR waves I, II, III, IV and V. The images for KM, KM plus DMSO, and KM plus SAHA illustrate representative ABR waveforms measured 1 week after the end of 15 days of treatment. [file Image_1.TIF]
